# Supplementary material for: Giant Atrial Dilatation: Systematic Review of Reported Cases from the Last Decade and an Illustrative Case with Dysphagia and Severe Dysphonia
Source: J Clin Med. 2025 Nov 4;14(21):7832. doi: 10.3390/jcm14217832 (PMC12609978; doi:10.3390/jcm14217832)
Supplement: Supplementary file 1 [file jcm-14-07832-s001.zip › Supplementary File S1. PRISMA 2020 for Abstracts Checklist .pdf]

PRISMA

PRISMA 2020 for Abstracts Checklist

Section and Topic	Item #	Checklist item	Reported (Yes/No)
**TITLE**			
Title	1	Giant Atrial Dilatation: Systematic Review of Reported Cases from the Last Decade and an Illustrative Case with Dysphagia and Severe Dysphonia	Yes
**BACKGROUND**			
Objectives	2	The objective was to review recent evidence on giant atrial pathology—including giant left atrium, giant right atrium, and atrial appendage aneurysms—and to illustrate their clinical relevance through cases of symptomatic extracardiac compression.	Yes
**METHODS**			
Eligibility criteria	3	Case reports and series of giant atrium (left, right, or appendage aneurysm) in humans, published in the last 10 years, in free full-text format. Reviews without primary cases, animal studies, abstracts without full text, and duplicates were excluded	Yes
Information sources	4	PubMed was searched on 15 September 2025 using the terms "giant atrium," "giant left atrium," "giant right atrium," and "atrial appendage aneurysm." Filters: human studies, last 10 years, free full text.	Yes
Risk of bias	5	No formal risk of bias assessment was performed, as only case reports and small series were included	No
Synthesis of results	6	Data were narratively synthesized and supported with descriptive statistics, figures, and tables. No meta-analysis was feasible due to heterogeneity.	Yes
**RESULTS**			
Included studies	7	A total of 21 publications reporting 24 individual cases were included. Patient age ranged from 22 to 89 years; the majority were female and had rheumatic mitral valve disease..	Yes
Synthesis of results	8	The most frequent manifestations were dyspnea, atrial fibrillation, and thromboembolism. Dysphagia and airway obstruction represented less common but clinically significant extracardiac complications. Most patients were managed conservatively, though surgical reduction or aneurysm resection was performed in selected symptomatic cases. Outcomes varied, with early deaths in some conservatively and surgically treated patients.	Yes
**DISCUSSION**			
Limitations of evidence	9	The available evidence is limited to case reports and small series, with inconsistent definitions of "giant" atrium and incomplete follow-up data, precluding quantitative synthesis.	Yes
Interpretation	10	Giant atrial pathology, although rare, carries significant hemodynamic, arrhythmic, and extracardiac risks. Early recognition and individualized management are essential, particularly in cases with compressive symptoms or thromboembolic complications.	Yes
**OTHER**			
Funding	11	This review was supported by Victor Babes University of Medicine and Pharmacy, Timisoara, which covered publication costs.	No

PRISMA 2020 for Abstracts Checklist

Section and Topic	Item #	Checklist item	Reported (Yes/No)
Registration	12	The review protocol was not registered in an international database.	Yes

From: Page MJ, McKenzie JE, Bossuyt PM, Boutron I, Hoffmann TC, Mulrow CD, et al. The PRISMA 2020 statement: an updated guideline for reporting systematic reviews. BMJ 2021;372:n71. doi: 10.1136/bmj.n71. This work is licensed under CC BY 4.0. To view a copy of this license, visit https://creativecommons.org/licenses/by/4.0/
