# Supplementary material for: Giant Atrial Dilatation: Systematic Review of Reported Cases from the Last Decade and an Illustrative Case with Dysphagia and Severe Dysphonia
Source: J Clin Med. 2025 Nov 4;14(21):7832. doi: 10.3390/jcm14217832 (PMC12609978; doi:10.3390/jcm14217832)
Supplement: Supplementary file 1 [file jcm-14-07832-s001.zip › Supplementary File S2. PRISMA 2020 Checklist.pdf]

PRISMA 2020 Checklist

Section and Topic	Item #	Checklist item	Location where item is reported
**TITLE**			
Title	1	Giant Atrial Dilatation: Systematic Review of Reported Cases from the Last Decade and an Illustrative Case with Dysphagia and Severe Dysphonia	Page 1
**ABSTRACT**			
Abstract	2	See the PRISMA 2020 for Abstracts checklist.	Page 1
**INTRODUCTION**			
Rationale	3	The introduction explains that giant atrial dilatation, particularly giant left atrium, is a rare but clinically significant condition. It has traditionally been linked to rheumatic mitral valve disease but is increasingly recognized in other settings. The rationale is to highlight its major complications (atrial fibrillation, thromboembolism, extracardiac compression) and the lack of consensus on optimal management, justifying a systematic review of recent evidence.	Page 2
Objectives	4	To systematically review reported cases of giant atrial dilatation (GLA, GRA, atrial appendage aneurysms) from the last decade and to illustrate clinical relevance with an institutional case of symptomatic extracardiac compression.	Page 2
**METHODS**			
Eligibility criteria	5	The review included case reports and case series from the last 10 years that provided original clinical data on giant atrial dilatation. Excluded were reviews without patient data, animal studies, abstracts without full text, and duplicates. Studies were grouped descriptively for synthesis.	Page 2
Information sources	6	The sole information source was PubMed, searched on September 15, 2025, using the terms “giant atrium,” “giant left atrium,” “giant right atrium,” and “atrial appendage aneurysm,” limited to human, free full-text articles published in the last 10 years.	Page 2
Search strategy	7	The search strategy involved a structured PubMed query on September 15, 2025, with the terms “giant atrium,” “giant left atrium,” “giant right atrium,” and “atrial appendage aneurysm.” Filters included human studies, free full-text, and publication within the last 10 years. This search returned 93 records, of which 21 were retained after screening.	Page 3
Selection process	8	Two reviewers independently screened titles and abstracts, followed by full-text assessment to determine eligibility. Disagreements were resolved by cross-checking. A total of 21 publications describing 24 cases were retained. No automation tools were applied.	Page 2-3
Data collection process	9	Data were extracted independently by two reviewers from each eligible report, then cross-checked for accuracy. Information collected included demographics, clinical presentation, comorbidities, diagnostic methods, atrial size, arrhythmias, complications, treatment strategies, and outcomes. No automation tools were used, and no additional data were obtained from study investigators.	Page 2-3
Data items	10a	Outcomes extracted included presenting symptoms, arrhythmias, hemodynamic complications (e.g., pulmonary hypertension), extracardiac manifestations, diagnostic findings (atrial size and imaging results), treatment strategies, and clinical outcomes such as complications, postoperative course, and survival.	Page 2-3
	10b	Besides outcomes, data items extracted included patient demographics (age, sex), comorbidities, prior cardiac surgery, atrium type involved, left ventricular ejection fraction, and presence of intracavitary thrombus. Assumptions were not explicitly reported for missing data, and in some cases information was marked as “not specified.”	Page 3
Study risk of bias assessment	11	No formal risk of bias tool was applied to the included case reports and series. Instead, the authors qualitatively acknowledged the limitations of available evidence, noting risks of selection and reporting bias due to database restriction, inclusion of only open-access English studies, and the reliance on case reports with heterogeneous reporting.	Page 2-3
Effect measures	12	No effect measures were specified because no quantitative synthesis was conducted. Results were summarized descriptively using means, medians, ranges, and frequencies of clinical features, complications, and outcomes.	Page 2-3
Synthesis methods	13a	Studies were included in the synthesis if they reported original patient-level data on clinical presentation, diagnostics, management, or outcomes of giant atrial dilatation. Eligible studies were narratively synthesized and compared with the institutional case.	Page 2-3
	13b	Data were organized into structured tables, with missing values recorded as “ns” or “na.” No statistical conversions or imputations were applied;	Page 3

Section and Topic	Item #	Checklist item	Location where item is reported
		information was synthesized as reported in the original case reports.	
	13c	Results were displayed in structured tables and visually summarized with charts and diagrams, including a study selection flowchart, demographic distribution graphs, symptom spectrum charts, and a boxplot of atrial dimensions.	Page 2-3
	13d	Results were synthesized narratively, highlighting recurring clinical and diagnostic patterns across the included cases. No meta-analysis was conducted due to heterogeneity and the descriptive nature of the data.	Page 2-3
	13e	Heterogeneity was assessed descriptively across multiple dimensions: demographics, clinical manifestations, atrial type involved, arrhythmic and thromboembolic complications, and treatment strategies. No statistical methods were applied, but variability was highlighted through tables, charts, and comparison with an institutional illustrative case.	Page 2-3
	13f	No sensitivity analyses were conducted. The review relied entirely on descriptive synthesis of case reports and series, and robustness of results was not formally assessed.	Page 2-3
Reporting bias assessment	14	The authors did not conduct a formal reporting bias assessment. However, they recognized the likelihood of selection and reporting biases due to restriction to a single database (PubMed), inclusion only of English-language and open-access full texts, reliance on case reports prone to selective publication, and frequent absence of long-term follow-up in the included studies.	Page 2-3
Certainty assessment	15	No formal certainty assessment tool was used. The certainty of evidence was considered low, given reliance on case reports and small series, heterogeneous reporting, and incomplete follow-up, limiting generalizability and confidence in the findings.	Page 2-3
**RESULTS**			
Study selection	16a	The PubMed search identified 93 records. After screening titles, abstracts, and full texts, 21 publications were included, reporting 24 cases. A study selection flow diagram (Figure 1) illustrates this process.	Page 3
	16b	Studies were excluded if they were reviews without primary cases, animal studies, abstracts without full text, or duplicates. Specific excluded studies were not individually cited in the article.	Page3
Study characteristics	17	Twenty-one publications reporting 24 patients with giant atrial dilatation were included. All were case reports or small series, spanning 2015–2024. Ages ranged from 22 to 89 years (mean 58.5, median 60), with a predominance of females (15 women, 8 men, 1 not specified). Clinical presentations included dyspnea, palpitations, edema, chest pain, and compressive symptoms (dysphagia, stridor, hoarseness). Diagnostics were mainly echocardiography, complemented by CT and MRI. Reported atrial dimensions reached up to 20.6 cm (left) and 13.3 cm (right). Arrhythmias, especially atrial fibrillation, were frequent (50%). Treatments varied between conservative therapy (anticoagulation, diuretics, rate control) and surgery (atrial reduction, reconstruction, appendage resection, valve surgery). Outcomes ranged from early death to survival beyond 10 years. Full study characteristics are tabulated in Appendix A, with references [4–34]	Page3-6
Risk of bias in studies	18	No formal risk of bias assessment tools were applied. Most included studies were single-patient case reports with incomplete reporting, variable definitions of “giant” atrium, and heterogeneous outcome data. These characteristics limit quality, increase susceptibility to publication and reporting bias, and prevent formal grading of study reliability.	Page3-8
Results of individual studies	19	Results for each individual case are provided in Appendix A (Table A1)<br><br>giant atrium, including demographics, symptoms, comorbidities, diagnostic modalities, atrial size, arrhythmias, treatments, complications, and outcomes. For example, one 45-year-old female [21] presented with dyspnea and palpitations, had a giant left atrium, atrial fibrillation, and was managed conservatively; she survived at least six months. Another patient, a 42-year-old male [23], underwent surgical resection but died 202 days later due to complications. These structured details allow case-by-case interpretation of outcomes.	Page 14-18
Results of syntheses	20a	Across all cases, patients were typically middle-aged or older women with rheumatic mitral disease. The risk of bias was high due to reliance on anecdotal reports with incomplete follow-up. Nevertheless, consistent patterns were observed: high prevalence of atrial fibrillation (50%), frequent pulmonary hypertension, and occasional but clinically decisive extracardiac compression.	Page3-8
	20b	No formal statistical meta-analysis was possible. Descriptive summaries included: mean atrial diameter 134 mm (up to 206 mm), atrial fibrillation in 12/24 patients (50%), intracavitary thrombus in 7/24 (29%). No pooled effect sizes were calculated.	Page3-8

PRISMA 2020 Checklist

Section and Topic	Item #	Checklist item	Location where item is reported
	20c	Heterogeneity arose from differences in patient age, comorbidities, atrial type involved (left, right, biatrial), diagnostic techniques, treatment approaches, and completeness of outcome data. Variability was explored descriptively, not statistically.	Page 5-7
	20d	No sensitivity analyses were performed.	Page 3-8
Reporting biases	21	No formal reporting bias assessment was performed. However, the review acknowledges a high likelihood of reporting bias, since only English-language, open-access, PubMed-indexed case reports were included, and such reports tend to overrepresent unusual or severe presentations.	Page 7-8
Certainty of evidence	22	No structured certainty assessment was conducted. Certainty was considered low, due to reliance on case reports and small series, heterogeneity in definitions and outcomes, incomplete follow-up, and absence of comparative studies. Conclusions should therefore be interpreted with caution.	Page 3-8
**DISCUSSION**			
Discussion	23a	The review interprets findings as confirming that giant atrial dilatation, though uncommon, has major clinical implications. It highlights the predominance in women with rheumatic mitral disease, the frequent complications (atrial fibrillation, thromboembolism, pulmonary hypertension), and the occasional but decisive extracardiac compressive symptoms.	Page 10-12
	23b	The evidence base is limited by reliance on case reports and small series, which are anecdotal and prone to selective reporting. Definitions of “giant atrium” were inconsistent, especially for right atrium and appendage aneurysms, and many reports lacked comprehensive follow-up or outcome data. These limitations reduce generalizability and weaken the strength of conclusions.	Page 10-12
	23c	The review process was limited by searching only one database (PubMed), restricting inclusion to English-language and open-access studies, and not applying formal tools for risk of bias or certainty assessment. Additionally, no efforts were made to obtain unpublished or non-indexed data, which may have introduced further selection bias	Page 10-12
	23d	The findings imply that clinicians should maintain a high index of suspicion for giant atrium in patients with atypical compressive symptoms and cardiomegaly. Management should be individualized, but earlier referral may improve outcomes. Future research should aim at standardizing definitions, surgical indications, and outcome reporting.	Page 10-12
**OTHER INFORMATION**			
Registration and protocol	24a	The review protocol was not registered in an international database.	Page 12-13
	24b	The review was conducted according to a predefined protocol based on PRISMA guidelines.	-
	24c	No protocol amendments were made, and any future changes will be reported transparently.	-
Support	25	The review was supported by Victor Babes University of Medicine and Pharmacy Timisoara, which covered publication costs. The funder had no role in the design, conduct, analysis, or reporting of the review.	Page 13
Competing interests	26	The authors declare no competing interests.	Page 13
Availability of data, code and other materials	27	All original data extracted for the review are included within the article and its appendix. No additional code or analytic datasets were generated. Further information can be obtained from the corresponding authors.	Page 13

From: Page MJ, McKenzie JE, Bossuyt PM, Boutron I, Hoffmann TC, Mulrow CD, et al. The PRISMA 2020 statement: an updated guideline for reporting systematic reviews. BMJ 2021;372:n71. doi: 10.1136/bmj.n71. This work is licensed under CC BY 4.0. To view a copy of this license, visit https://creativecommons.org/licenses/by/4.0/
